# Development of an automatic integrated gene detection system for novel severe acute respiratory syndrome-related coronavirus (SARS-CoV2)

**DOI:** 10.1080/22221751.2020.1782774

**Published:** 2020-07-03

**Authors:** Yuchang Li, Jing Li, Ying Zhang, Lizhong Dai, Lin Li, Juan Liu, Sen Zhang, Xiaoyan Wu, Yi Hu, Chengfeng Qin, Tao Jiang, Xiaoping Kang

**Affiliations:** a State Key Laboratory of Pathogen and Biosecurity, Beijing Institute of Microbiology and Epidemiology, The Academy of Military Medical Science, Beijing, China; b Sansure Biotech Inc., Changsha, China

**Keywords:** SARS-CoV2, automatic integrated gene detection system, qRT-PCR, COVID-19, rapid detection

## Abstract

In December 2019, Wuhan, China suffered a serious outbreak of a novel coronavirus infectious disease (COVID) caused by novel severe acute respiratory syndrome-related coronavirus (SARS-CoV 2). To quickly identify the pathogen, we designed and screened primer sets, and established a sensitive and specific qRT-PCR assay for SARS-CoV 2; the lower limit of detection (LOD) was 15 (95% CI: 9.8–21) copies per reaction. We combined this qRT-PCR assay with an automatic integration system for nucleic acid extraction and amplification, thereby establishing an automatic integrated gene detection system (AIGS) for SARS-CoV 2. Cross reactive analysis performed in 20 other respiratory viruses and 37 nasopharyngeal swabs confirmed a 100% specificity of the assay. Using two fold diluted SARS-CoV 2 culture, the LOD of AIGS was confirmed to be 365 copies/ml (95% CI: 350–375), which was Comparable to that of conventional qRT-PCR (740 copies/ml, 95% CI: 690–750). Clinical performances of AIGS assay were assessed in 266 suspected COVID-19 clinical respiratory tract samples tested in parallel with a commercial kit. The clinical sensitivity of the AIGS test was 97.62% (95% CI: 0.9320–0.9951) based on the commercial kit test result, and concordance analysis showed a high agreement in SARS-CoV-2 detection between the two assays, Pearson R was 0.9623 (95% CI: 0.9523–0.9703). The results indicated that this AIGS could be used for rapid detection of SARS-CoV 2. With the advantage of simple operation and less time consuming, AIGS could be suitable for SARS-CoV2 detection in primary medical institutions, thus would do a great help to improve detection efficiency and control the spread of COVID-19.

At the end of December 2019, an outbreak of unexplained pneumonia occurred in Wuhan, China [1–3]. The pathogen was identified as a novel coronavirus, SAR-CoV 2[4]. Sequence identity between this pathogen and SARS-CoV is ∼80% [5–8]. SARS-CoV 2 is highly contagious, with the number of cases increasing rapidly in just 1 month, and the number of cases in China exceeded 80,000 at the end of March 2020. Although the fatality rate is around 2%, the severity rate can reach 25%, seriously endangering people’s health, and affecting their normal work and lives [9–16]. Rapid screening of suspected patients is a prerequisite for prevention and control of SARS-CoV 2 infection.

According to the guidelines for the prevention and control of coronavirus infectious disease (COVID-19) published by the national center for disease control (CDC) [17], nucleic acid testing is the standard method for the diagnosis of SARS-CoV 2 infection. Due to the lack of specialized nucleic acid extraction and amplification laboratories in primary institutions, testing has primarily been conducted by CDC sections and tertiary hospitals. With the further expansion of the epidemic, it is difficult for the provincial CDCs and tertiary hospitals to finish all tests in time. Therefore, it is urgently necessary to develop simple sample processing and a nucleic acid testing system for application in primary medical institutions, enabling clinicians to quickly triage patients, and effectively prevent and control the epidemic.

In the early stages of our pathogen identification efforts, we designed three sets of primers and probes based on the genome sequence of SARS-CoV 2, identified an S gene primer set with high sensitivity, and established a qRT-PCR assay capable of identifying SARS-CoV 2 infection. In this study, we combined this qRT-PCR assay with a new automatic nucleic acid detection system. The resultant system can detect SARS-CoV 2 directly in original samples, enabling its application in primary medical institutions.

## 1. Materials and methods

### 1.1 Design of primers and probes

Based on the genome sequence of SARS-CoV2, we designed primers and probes for the non-coding region, N gene, and S gene. The RNaseP gene was used as the internal reference gene for qRT-PCR. Primer and probe sequences are shown in supplemental tablet 1; the oligo nucleotides were synthesized by Sango (Shanghai, China).

### 1.2 Clinical samples and virus culture

We obtained a total of 266 clinical samples from 230 patients suspected to have COVID-19, mainly from the CDC of Hubei province and Huoshenshan Hospital. These included three lung lavage samples, 22 sputum samples, 230 throat swabs, eight nose swabs, and three blood samples.

Thirty Seven additional nasopharyngeal swabs from patients known to be positive for other respiratory disease associated virus were used to establish clinical specificity of the conventional qRT-PCR and AIGS assay, including 12 swab samples for infuenza A, eight samples for influenza B, five samples for human coronaviruses 229E, seven samples for OC43, five samples for adenovirus.

All patients provided written informed consent, which was approved by the hospital ethics committee.

Supernatants of inactivated SARS-CoV2 cultures V34 strain (8 × 10^6^ pfu/ml) were collected and used to determine the sensitivity of qRT-PCR. For determination of specificity, supernatants were also collected for 20 species of respiratory tract infection associated viruses including adenovirus types 3, 4, 7,11, 14 and 55; influenza A virus subtypes H1N1, H3N2, H7N9; influenza B virus, parainfluenza virus types 1, 2 and 3; Entero virus EV 71 and CA16, respiratory syncytial virus, inactivated SARS-CoV, coronavirus 229E, OC43and inactivated MERS-CoV.

### 1.3 RNA extraction

RNA was extracted from 200 µL each clinical sample or viral culture using the Viral RNA mini kit (QIAGEN, Hilden, Germany). A total of 50 µL RNA were eluted from each extracting colummn for each sample.

### 1.4 Quantitative RT–PCR assay

The qRT-PCR reaction contained 5 µl RNA, 5 µl of 4× TaqMan Fast Virus 1-step mix (Applied Biosystems, Vilnius, Lithuania), 1 µl forward primer (10 µM), 1 µl reverse primer (10 µM), 0.5 µl probe (10 µM), and 7.5 µl sterile deionized water; final volume was 20 µl. Reactions were performed in a LightCycler 480 Real Time PCR instrument (Roche Diagnostics, Mannheim, Germany). Amplification conditions were as follows: reverse transcription at 50°C for 5 min; pre-denaturation at 95°C for 10 s; and 40 cycles of PCR amplification consisting of denaturation at 95°C for 5 s, annealing at 60°C for 30 s, and fluorescence measurement. We also tested these clinical samples using the SARS-CoV 2 nucleic acid detection kit (Sansure Biotech, Changsha, China), which has been authorized for clinical use by the FDA (China). The reaction mixture contained 26 µl buffer, 4 µl enzyme reaction solution, and 10 µl RNA template. Reaction conditions were as follows: reverse transcription at 50°C for 30 min, pre-denaturation at 95°C for 1 min, and 45 cycles of PCR amplification consisting of denaturation at 95°C for 15 s, annealing at 60°C for 30 s, and fluorescence measurement.

### 1.5 Automatic integrated gene detection and analysis system (AIGS)

**Figure 1.**
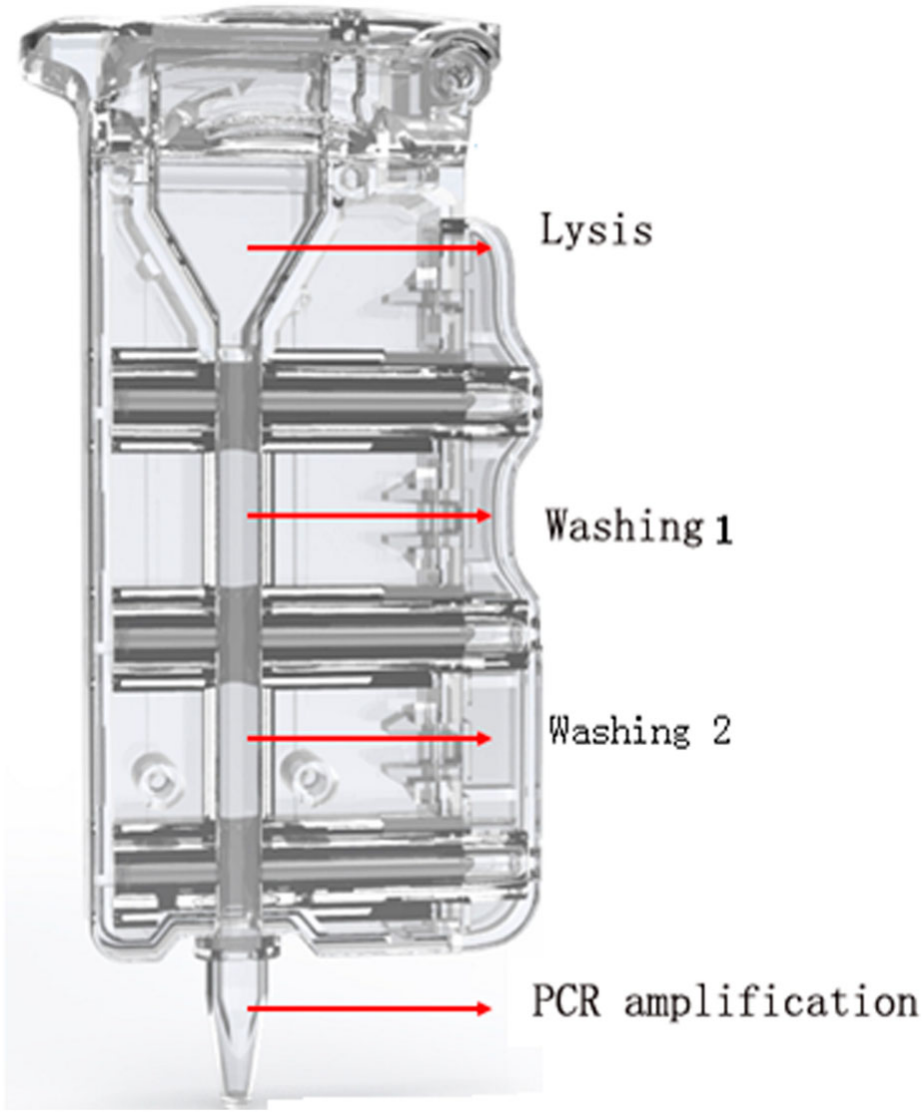
Schematic of AIGS cartridge. The cartridge consists of the lysis area, washing area 1, washing area 2, and PCR amplification area; adjacent areas are separated by silicone oil and a plunger seal. The virus was lysed with detergent in the lysis area, and DNA/RNA bound to the magnetic beads under the high-salt conditions in the lysate. The magnet inside the instrument attracted the magnetic beads and pulled them into washing areas 1 and 2 for RNA/DNA extraction. Finally, the magnetic beads were dragged into the PCR amplification area for nucleic acids amplification and detection.

The AIGS consisted of cartridges and a device. Reagents for nucleic acid purification and qRT-PCR amplification were placed in the cartridges in advance. The structure of the cartridge (Figure 1) consists of the lysis area, washing area 1, washing area 2, and PCR amplification area; adjacent areas are separated by silicone oil and a plunger seal.

**Figure 2.**
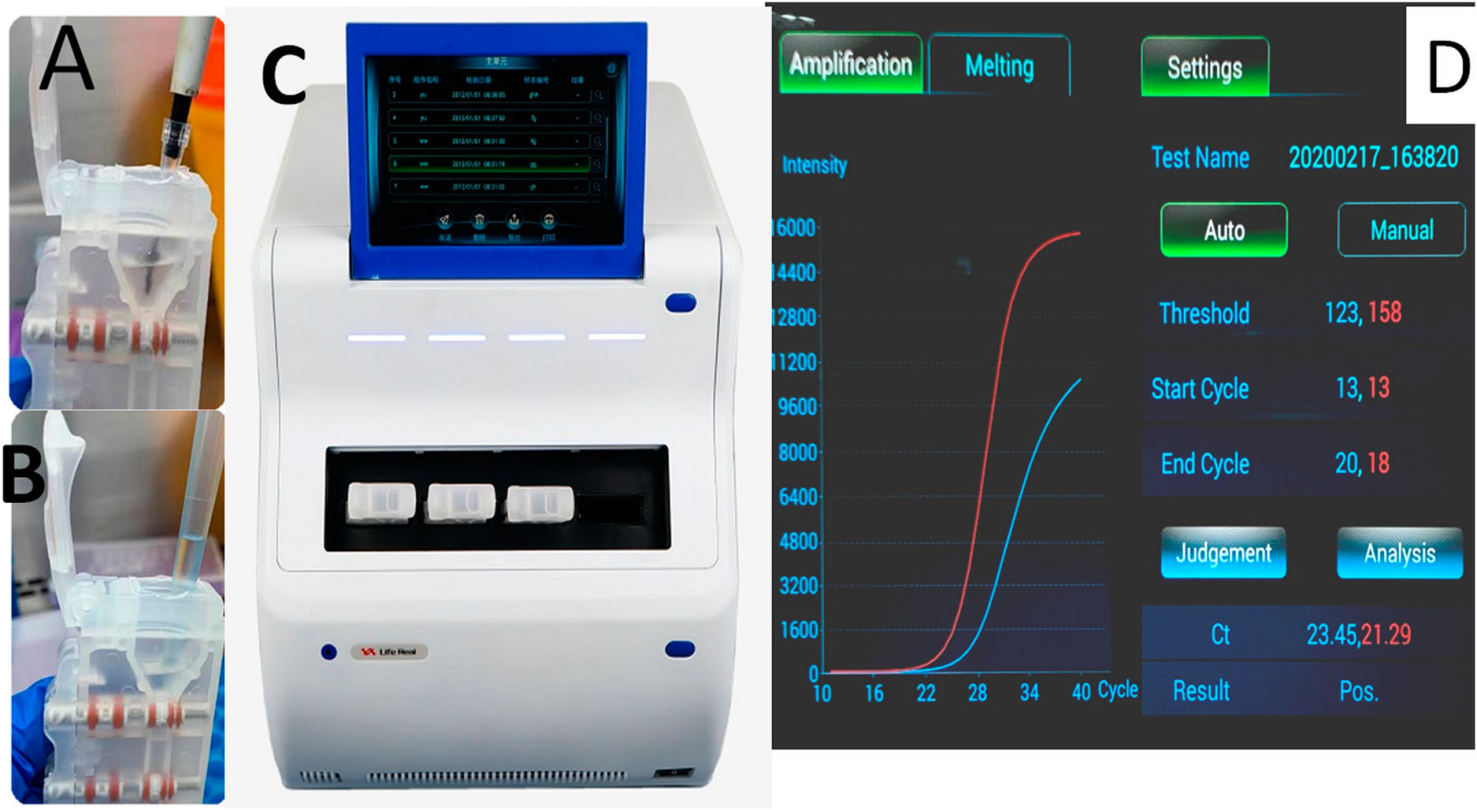
AIGS flow chart. A: add 10ul magnetic beads; B: add 200ul sample to each cartriadge; C: Inserting the cartridge into detection site; D: result analysis. Thermal cycling conditions were as follows: reverse transcription, 56°C for15 min; initial denaturation, 94°C for1 min; and 40 cycles of 95°C for 10 s and 58°C for 30 s. The amplification curve was displayed on the screen in real time while the amplification programme ran.

The sample tubes containing clinical specimens were placed in a water bath at 56°C for 30 min to inactive the virus, then 200 µl sample was taken out from the tube and applied to the AIGS for detection. The automated nucleic acid extraction and detection process in the AIGS proceeded as follows: 10 µl magnetic beads and 200 µl inactivated sample were added to the lysis area, and after mixing, the cartridge was inserted into the automatic nucleic acid detection and analysis device (LifeReady1000, Hangzhou Lifereal Biotechnology, Hangzhou, China) for testing (Figure 2). The virus was lysed with detergent in the lysis area, and viral protein was separated from DNA/RNA. DNA and RNA bound to the magnetic beads under the high-salt conditions in the lysate. The magnet inside the instrument attracted the magnetic beads and pulled them into washing areas 1 and 2 to remove attached protein, fibre, and other impurities. Finally, the magnetic beads were dragged into the PCR amplification area and eluted at 95°C to promote release of nucleic acids. RNA-direct Real Time PCR Master Mix (qRT-101; Toyobo, Osaka, Japan), CoV p3 set (forword primer and reverse primer, 16 pmol each; probe, 8 pmol), and internal reference primer and probe were placed in the amplification area in advance. Thermal cycling conditions were as follows: reverse transcription, 56°C for15 min; initial denaturation, 94°C for 1 min; and 40 cycles of 95°C for 10 s and 58°C for 30 s. The amplification curve was displayed on the screen in real time while the amplification programme ran and the total detection time was about 80 min. This detection system was double-channel as follows: FAM-labeled coronavirus detection probe and ROX-labeled internal reference detection probe. If the CT value of FAM channel was less than 40, the sample was determined to be positive; otherwise, the sample was considered negative.

### 1.6 Statistical analysis

The analytical sensitivity (SARS-Cov-2 copy number at a 95% confidence interval) was calculated with probit analysis, using the Graphpad 5 statistical software, on the basis of results obtained by several replicates of serial dilutions of the SARS-Cov-2 culture and recombinant T7 transcript RNA containing specific target genes quantification standard. The clinical sensitivity analysis and evaluation of the concordance between results obtained with AIGS and the commercial 2019-nCoV nucleic acid detection kit was performed using GraphPad Prism 5 chi-square test and Spearman test, respectively.

## 2. Results

### 2.1. Cov-p3 Primer set has high sensitivity and specificity for SARS-CoV 2 detection

Nucleic acids from 20 species of respiratory associated viruses including SARS-CoV, MERS-CoV, OC229E, OC43, adenovirus types 3, 4, 7, 11, 14 and 55; influenza A virus subtypes H1N1, H3N2, and H7N9; influenza B virus, parainfluenza virus type 1, 2 and 3, and respiratory syncytial virus, enterovirus EV71 and CA16 were used to evaluate specificity. None of the three primer sets including Cov-p1, Cov-p2, and Cov-p3 cross-reacted with any of the 20 species of respiratory associated viruses.

Because we could initially determine the sensitivity of the primers by comparing differences in their CT values, we used two patient samples as RNA templates to rapidly select the most sensitive primer for SARS-CoV 2 detection. The results revealed that Cov-p3 yielded the lowest CT values for both samples, indicating that the Cov-p3 was the most sensitive primer for the detection of SARS-CoV 2 in both samples (Table 1).

**Table 1.** Sensitivity comparison of three primer sets.

	CT value	
Primer set	Patient 1	Patient 2
CoV-Primer 1	37.49	34.20
CoV-Primer 2	36.12	33.15
CoV-Primer 3	34.81	31.78

**Figure 3.**
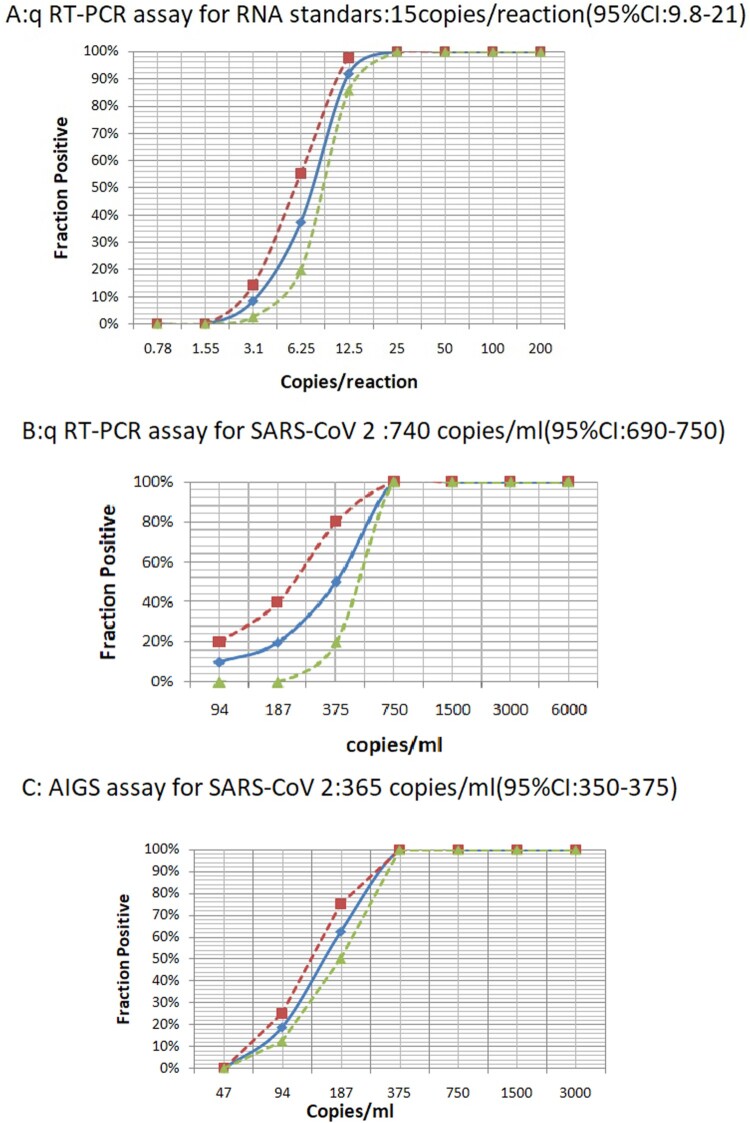
LOD test by conventional qRT-PCR and AIGS. A: Series of two fold diluted T7 transcripted RNA containing specific target genes were applied for conventional qRT-PCR assay. B: Series of two fold diluted RNA from cultured SARS-CoV 2 culture were applied for conventional qRT-PCR assay. C: Series of two fold diluted SARS-CoV 2 culture were applied for AIGS assay. The x-axis shows input RNA copies per reaction or virus per microtiter. The y-axis shows positive results in all parallel reactions performed, squares are experimental data points resulting from replicate testing of given concentrations (x-axis) in parallels assays (eight replicate reactions per point). Limits of detection are given in the panels headings. The inner line is a probit curve (dose-response rule). The outer dotted lines are 95% Confidence Interval.

To accurately quantify the detection sensitivity of Cov-p3, we performed ten- fold serial dilutions of T7 transcript RNA containing specific target genes and used them as templates in qRT-PCR, which demonstrated that the LOD was about 1–100 copies per reaction (Supple Figure 1). We then performed qRT–PCR on 2-fold serial dilutions. Two independent experiments with 8 replicates per sample were conducted at concentrations around the detection end point determined in the previous dilution experiments. As shown in Figure 3(a), Cov-p3 had a sensitivity of 14.8 copies/reaction (95% confidence intervals: 9.8–21).

### 2.2. Sensitivity and specificity of AIGS

RNAs from a SARS-CoV 2 culture (8 × 10^6^ pfu/ml) was quantified by qRT-PCR using recombinant T7 transcript RNA containing specific target genes quantification standard. From the standard curve, it was calculated that 1 pfu SARS-CoV 2 culture generated 455.6 gene copies of SARS-CoV 2.

For the LOD determination of AIGS, initially we performed experiments on 10-fold serial dilutions of the SARS-CoV 2 culture, which demonstrated that the LOD was about 50–2000 copies/ml.

Two independent experiments with 8 replicates per sample were conducted at concentrations around the detection end point determined in the previous dilution experiments. Eight concentrations and 200 µl aliquots of each sample were used to evaluate the sensitivity of the AIGS. At the same time, nucleic acids from an equivalent amount of diluted viruses were extracted and subjected to conventional qRT-PCR amplification. The results revealed the LOD from replicate tests was 365 copies/ml (95% CI: 350–375) for the AIGS assay and 740 copies/ml (95% CI: 690–750) for conventional qRT-PCR, as shown in Figure 3(b,c). This indicated that although the LOD per reaction of AIGS was lower than that of conventional qRT-PCR (73 copies/reaction versus 15 copies/reaction), AIGS used a larger volume of samples than the conventional qRT-PCR and achieved comparable LOD to the conventional qRT-PCR for the original samples test.

For the specificity detection, we used inactivated 37 clinical swab samples positive for other respiratory disease associated viruses and 20 respiratory infection associated virus culture including SARS-CoV, MERS-CoV, OC229E, OC43, adenovirus type 3, 4, 7, 11, 14 and type 55, influenza A virus H1N1, H3N2, and H7N9, influenza B virus, parainfluenza virus type 1, type 2, and type 3, and respiratory syncytial virus, enterovirus EV71, CA16 as templates to confirm the specificity of the AIGS. We observed no cross-reaction for any of the inactivated viruses, indicating that the AIGS was 100% specificity with 95% confidence intervals for SARS-CoV 2 detection.

### 2.3. Performance evaluation of AIGS for clinical samples

We collected 266 clinical samples from 230 suspected COVID-19 patients and subjected them to AIGS detection. In parallel, we compared the results with those of the commercial 2019 nCoV nucleic acid detection kit (Sansure, Changsha). The results were shown in Table 2.

**Table 2.** Validation of the AIGS assay using clinical samples.

	Commercial kit			
		P	N	Total
AIGS	P	123	2	125
	N	3	138	141
	Total.	126	140	266

Note: L: Lung lavage fluid; P: Pharyngeal swab; N: Nasal swabs; S: Sputum; B: Blood. Entries in the columns corresponding to each assay indicate the number of positive samples detected.

In total, 230 pharynx swabs, three samples of lung lavage, eightnose swabs, 22 sputum samples, and three blood samples were tested using the commercial 2019 nCoV nucleic acid detection kit, which identified 126 positive samples and 140 negative samples. By contrast, the AIGS identified 125 positive samples and 141 negative samples. The clinical sensitivity of this AIGS was 97.62% (95% CI: 0.9320–0.9951) based on the commercial 2019 nCoV nucleic acid detection kit, and concordance analysis showed a high agreement in SARS-CoV-2 detection between the two assays, Pearson R was 0.9623(95% CI: 0.9523–0.9703).

Among the 266 clinical samples, three negative samples tested by AIGS were identified as positive by the commercial kit, and two positive samples tested by AIGS were identified as negative by the commercial kit. Then we tested IgM antibodies for SARS-CoV 2 in the sera from the five inconsistent patients, all of the five sera samples were positive, indicating that all the five inconsistent patients were positive for SARS-CoV 2 infection.

We also compared the results among different types of samples from the same patient, including lung lavage, sputum, throat swabs, and blood samples. Lung lavage contained the highest concentration of nucleic acid, followed by sputum and pharynx swab; blood samples yielded a much lower positive rate than other sample types (Table 3), suggesting that samples from the lower respiratory tract, such as lung lavage and sputum, are optimal for nucleic acid detection to identify SARS-CoV 2 infection.

**Table 3.** Comparison of qPCR results from different specimens.

Sample type	CT value		
	Patient 1	Patient 2	Patient 3
L	29.10	24.30	26.39
S	29.62	29.09	30.62
P&N	36.12	30.91	0
B	0	0	0

Note: L: Lung lavage fluid; P&N: Pharyngeal swab and Nasal swabs; S: Sputum; B: Blood.

## 3 Discussion

qPCR results reflect the presence of viral nucleic acid in samples; accordingly, this method is well suited for early diagnosis of virus infection. At present, qPCR is the most important detection method for clinical diagnosis of infectious diseases [18–21].

SARS-CoV2 was first reported in late December 2019 [1], and the first commercial kit for nucleic acid detection was authorized on January 26, 2020. As early as January 5, we started performing test to identify suspected cases of COVID. To develop a suitable qRT-PCR method, we designed and screened sensitive primer sets for qRT-PCR assays. The LOD could reach 15 copies per reaction (95% CI: 9.8–21) with no cross-reaction with other respiratory disease associated virus. Testing of 171 clinical samples (41 positive and 130 negative for SARS-CoV 2) showed that the accuracy of the test was comparable to that of commercial kits (data not shown).

To establish a simpler and more practical detection system for use in primary medical institutions, we pre-fixed the CoV-p3 primers in the AIGS cartridge, which allowed an automatic integrated nucleic acid detection system of SARS-CoV2. The LOD of AIGS could reached 365 copies/ml (95% CI: 350–375) for original sample, which was similar to that of conventional qRT-PCR (740 copies/ml, 95% CI: 690–750). In single reactions, the LOD of AIGS was lower than that of conventional qRT-PCR (73 copies versus 15 copies per reaction), this might because AIGS integrated extraction and amplification and accommodated a larger sample size, all of the extracted nucleic acid was contained in the reaction cartridge used for amplification. By contrast, the conventional qRT-PCR required a separate extraction process, and the volume of RNA was limited to 5–10 µL, meaning that only 10% of extracted RNA could be added into the reaction tube. Thus, if volume consideration was disregarded, AIGS could have comparable sensitivity to that of conventional qRT-PCR for the original samples analysis. Since AIGS could detect SARS-CoV2 in original samples directly, it permited a more accurate description of the LOD in terms of copies per volume instead of copies per reaction.

We tested a variety of clinical samples, including lung lavage, sputum, nose swab, throat swab, and other types, and identified a total of 125 positive samples (CT value: 25.07–38.72) and 141 negative samples by AIGS. Compared with the commercial test, concordance rate was 97.62% for the positive samples and 98.5% for the negative samples. IgM test confirmed that all of the five inconsistent patients were positive for COVID-19, these results confirmed that the AIGS would be potentially useful for detect multiple types of respiratory tract samples in clinical use.

We also measured different types of samples from the same patients, including lung lavage, sputum, throat swabs, and blood. Of these four types of samples, lung lavage had the highest concentration of nucleic acid, followed by sputum, indicating that samples from the lower respiratory tract are optimal for nucleic acid detection of SARS-CoV 2.

For diagnostic detection of SARS-CoV2, the current commercial nucleic acid detection kits use conventional qRT–PCR methods that require inactivation and extraction procedures in specialized laboratories and work by trained professionals, as well as larger amounts of time [22–24]. More and more suspected cases have appeared around the world, leading WHO to characterize COVID-19 as a pandemic. By May 2020, the number of COVID-19 cases had exceeded 5 milion wordwide. It is urgent to carry out COVID-19 testing early in primary medical institutions. However, such institutions often cannot conduct qRT-PCR testing because they lack professional laboratories. Consequently, samples from suspected cases must be sent to other professional departments for testing, prolonging the time required for case confirmation and increasing the risk of further spread of the virus [25,26].

Our AIGS integrates nucleic acid extraction and PCR amplification, thus can be used to detect samples directly. According to the CDC’s 2019 guidelines for COVID diagnosis and treatment, the virus in clinical samples can be inactivated by incubation at 56°C for 30 min [12]. Therefore, for primary laboratories without a biosafety rating, a water bath combined with this AIGS could be used to test for COVID cases. Moreover, the cartridges of AIGS can be operated separately, so samples can be tested at any time without waiting, which is ideal for application in primary medical institutions.

Although the AIGS we used can only detect 4 samples at same time, eight and more detection sites of AIGS instruments are being developed at present, moreover, multiple detections can be performed since AIGS has five different fluorescence detection channels. So, combining more sites of instrument with multi channels detection, the AIGS can be expanded into more samples and more pathogens detection.

With its advantages of simple operation, rapid process, and high accuracy, this AIGS is potentially useful in primary medical institutions, and could thus play an important role in the prevention and control of SARS-CoV 2.

## Supplementary Material

Supplemental Material
